# Limited inclusion in clinical trials and access to poly (ADP-ribose) polymerase inhibitors in racial and ethnic minority patients with cancer: a narrative review

**DOI:** 10.1093/oncolo/oyag291

**Published:** 2026-07-25

**Authors:** Ashleigh Swearingen, Bala Audu, Ahmed Hamidu, Aisha Mustapha, Adekunle Oguntayo, Anisah Yahya, Abubakar Bello, Raleigh Butler, Darron Halliday, Saida Bowe, Scott Jordan, Karina Hew, Andre Pinto, Rosa Patricia Castillo, Isildinha M Reis, Sophia George, Matthew Schlumbrecht

**Affiliations:** Division of Gynecologic Oncology, Sylvester Comprehensive Cancer Center, Miami, FL 33136, United States; Department of Obstetrics, Gynecology, and Reproductive Sciences, University of Miami Miller School of Medicine, Miami, FL 33136, United States; Federal University of Health Sciences Azare (FUHSA), Azare, Nigeria; Ahmadu Bello University Teaching Hospital (ABUTH), Zaria, Nigeria; Ahmadu Bello University Teaching Hospital (ABUTH), Zaria, Nigeria; Ahmadu Bello University Teaching Hospital (ABUTH), Zaria, Nigeria; Ahmadu Bello University Teaching Hospital (ABUTH), Zaria, Nigeria; Federal University of Health Sciences Azare (FUHSA), Azare, Nigeria; University of the West Indies-Nassau, Nassau, Bahamas; University of the West Indies-Nassau, Nassau, Bahamas; University of the West Indies-Nassau, Nassau, Bahamas; Broward Health, Fort Lauderdale, FL 33316, United States; Department of Obstetrics and Gynecology, University of Florida-Jacksonville, Jacksonville, FL 32209, United States; Department of Pathology, University of Miami Miller School of Medicine, Miami, FL 33136, United States; Department of Radiology, University of Miami Miller School of Medicine, Miami, FL 33136, United States; Division of Biostatistics and Bioinformatics, Department of Public Health Sciences, University of Miami Miller School of Medicine, Miami, FL 33136, United States; Division of Gynecologic Oncology, Sylvester Comprehensive Cancer Center, Miami, FL 33136, United States; Department of Obstetrics, Gynecology, and Reproductive Sciences, University of Miami Miller School of Medicine, Miami, FL 33136, United States; Division of Gynecologic Oncology, Sylvester Comprehensive Cancer Center, Miami, FL 33136, United States; Department of Obstetrics, Gynecology, and Reproductive Sciences, University of Miami Miller School of Medicine, Miami, FL 33136, United States

**Keywords:** disparities, PARP inhibitor, global access, African ancestry, clinical trials

## Abstract

**Background:**

Poly ADP-ribose polymerase (PARP) inhibitors (PARPi) have revolutionized cancer care across multiple solid tumor types for patients with BRCA mutations and homologous recombination deficient malignancies. Sentinel trials for PARPi, however, have had limited participation from non-European populations, limiting interpretation of results, dosing strategies, and quality-of-life implications in these patients. Our objective was to review participation by race and ethnicity in sentinel PARPi trials across disease sites.

**Methods:**

A literature search was conducted using PubMed/MEDLINE. Core search terms included “PARP inhibitor” and the disease sites of interest (breast, ovary, prostate, and pancreas). A review of each sentinel trial was conducted utilizing data from ClinicalTrials.gov to obtain and/or confirm demographic information.

**Results:**

Sentinel PARPi trials across all disease sites included populations that were predominantly White. TRITON2 (prostate) had the highest proportion of Black participants (5.8%), while Black participation in breast and ovarian cancer trials was almost always <3%. Missing data on race and ethnicity was common, and highest in TRITON2 and TRITON3 (∼20%, prostate) and ARIEL3 (13.3%, ovary). Aggregation into an “other” category was highest in EMBRACA (17.2%). Hispanic ethnicity was reported in only 5 of the 14 trials.

**Conclusion:**

There is overwhelming underrepresentation of racial and ethnic minorities in sentinel PARPi trials. Systemic issues in accepted clinical trial infrastructure, including reporting practices, site selection, protocol biases, and access challenges are all likely etiologies. Addressing the complexity of inclusion in global oncology research is still a crucial need to ensure cancer care equity.

Implications for Practice The underrepresentation of marginalized populations in PARP-inhibitor (PARPi) trials creates a global access gap that undermines the equitable translation of precision oncology into clinical practice. Current treatment guidelines are derived from trials with populations overrepresented by European ancestry patients, leaving a significant disparity in our understanding of PARPi efficacy, safety, and quality of life effects in minority populations. Equitable expansion of PARPi trials to these patients globally requires the optimization of genomic testing infrastructure, more personalized inclusion/exclusion criteria, and the mitigation of prohibitive costs and regulatory practices that restrict access in low resource, but ancestrally rich, settings.

## Introduction

Clinical trials on novel therapeutics in oncology have vast underrepresentation of racial and ethnic marginalized populations relative to respective global cancer incidence.^1^ Despite global reach, sentinel poly ADP-ribose polymerase inhibitor (PARPi) trials for ovarian, breast, pancreatic, and prostate cancer have had unequal representation across race, ethnicity, and geography. Consequently, the translation of trial-derived data into clinical practice favors populations of European descent, limiting interpretation of results to the wider oncology community.

Poly ADP-ribose polymerases (PARP1 and PARP2, otherwise called PARP herein) facilitate repair of single-strand DNA and double strand breaks. Inhibitors of PARP are most efficient in tumors with homologous recombination deficiency (HRD), which are unable to repair double-strand DNA breaks, and thus leading to synthetic lethality.^2^ HRD is present in patients with germline or somatic mutations in the Fanconi Anemia and Homologous Repair pathways, such as BRCA1 and BRCA2. Originally discovered in the early 2000s through BRCA synthetic lethality screens,^3^ PARPi gained traction with phase 2 studies confirming the efficacy of olaparib maintenance therapy in patients with platinum-sensitive recurrent high-grade serous ovarian, fallopian tube, or primary peritoneal cancer, and leading to the first regulatory approval for this class of drugs.^4^^,^^5^ This paved the way for phase 3 sentinel trials such as SOLO-1, PRIMA, and ATHENA-MONO, which established frontline maintenance monotherapy approvals. A major critique of these studies, however, has been the low enrollment of Black, Asian, and Hispanic patients.^6–8^ Underrepresentation of non-White patients in clinical trial enrollment has been perpetuated as PARPi were found to have therapeutic benefit in other disease sites, and their utilization expanded, leading to the current landscape where treatment guidelines are informed almost exclusively by populations of European ancestry.

Gaps in representation, and trial population demographics that do not align with global cancer incidence rates, have been identified as systemic barriers to cancer care.^9^ The reasons for such gaps, however, are multifactorial and in many ways accentuated in research specifically related to PARPi use and eligibility. Limited genomic diversity from population based databases,^10^ impacted by unequal availability of next-generation sequencing and genetic testing, translates into poor understanding of the prevalence of HRD or germline and somatic BRCA mutations in minority patients.^11–14^ Overly restrictive inclusion criteria, such as exclusion of patients with HIV disease and other comorbidities, prevent enrollment of patients who would potentially derive benefit from treatment. Socioeconomic barriers, with inability to pay high drug costs, as well as limited research infrastructure and lack of homogeneity across national regulatory bodies, reduces global access, especially in low- and middle-income countries (LMIC).^15–19^

This review examines the distribution of minority participation in sentinel PARPi trials for ovarian, breast, prostate, and pancreatic cancers, and how limited participation of this group restrict applicability of scope of this class of drugs. It also highlights global factors influencing access to PARPi for non-European ancestry patients, considering systemic issues in the conduct of clinical trials, eligibility criteria, and structural global inequities which further exacerbate disparities.

## Methods

### Study design and objectives

The objective of this review is to identify racial and ethnic disparities in sentinel clinical trials evaluating PARPi as a single agent in ovarian, breast, prostate, and pancreatic cancers. Secondary objectives included an evaluation of trial eligibility criteria, global access to PARPi therapy, and inequities in germline and genomic testing as drivers for these disparities.

### Literature search strategy

A literature search was conducted using PubMed/MEDLINE without a specified date range. Core search terms (MeSH) included “PARP inhibitor,” “poly(ADP-ribose) polymerase inhibitor,” “olaparib,” “niraparib,” “rucaparib,” “talazoparib,” combined with disease-specific terms (“ovarian cancer,” “breast cancer,” “prostate cancer,” “pancreatic cancer”) and trial descriptors “phase II,” “phase III,” “randomized,” “clinical trial.” References were manually screened to identify sentinel trials, which are defined as studies that provide foundational evidence for the first regulatory approval of a drug class for a specific disease and establish a new standard of care within national or international guidelines. A review of each sentinel trial was conducted utilizing data from ClinicalTrials.gov to obtain and/or confirm demographic information.

## Results

### General patterns of representation in oncology clinical trials

Current enrollment does not reflect the real-world demographic epidemiology of cancer incidence or national/geographic populations. Khadraoui et al. reported on a Surveillance, Epidemiology, and End Results Program population of >500 000 women from 2004 to 2019 with cervical, ovarian, and endometrial cancer. Relative to the entire population in the United States, White women were adequately or overrepresented in clinical trials across all disease sites, while Asian and Hispanic women were underrepresented for all cancer types, and Black women underrepresented for ovarian cancer.^20^

An analysis of 230 landmark trials found that Black patients were enrolled at only 22% of their expected proportion and Hispanic patients at 44%, while Asian patients were over-represented at 438% of their expected proportion relative to US incidence.^21^ In such cases, disparities are often incorrectly attributed to patient hesitancy, but research shows that willingness to participate does not differ by race, and simple educational interventions increase interest across all groups.^22^^,^^23^

### Diverse populations may benefit from PARP inhibition

The Hereditary Breast and Ovarian Cancer Syndrome, driven primarily by mutations in the BRCA1 and BRCA2 genes, increases susceptibility to breast, ovarian, pancreatic, and prostate cancers.^24^ There are unique patterns within these diseases across populations of racial and ethnic minorities that speak to the importance of the inclusion of these patients in PARPi trials. For example, prostate cancer is the leading cancer for men of African descent in the United States, Caribbean, and Sub-Saharan Africa.^25^

In populations of women, Chapman-Davis et al. noted then when excluding Ashkenazi Jewish founder effects, the prevalence of BRCA mutations was lowest in non-Hispanic White women (non-Hispanic White 12.1%; Black 15.6%; Hispanic 14.8%; Asian at 12.7%).^26^ Women from the Bahamas with breast cancer have the highest known prevalence of BRCA mutations worldwide, with approximately 23% carrying a BRCA mutation (predominantly BRCA1).^27^ Across the Caribbean, which has a population that is predominantly Hispanic and/or Black, there are high, and variable rates, of BRCA1, BRCA2, and PALB2 mutations that predispose to early onset breast cancer.^28^ Clear BRCA founder effects have also been reported throughout Latin America, with significant variation by geography and ancestry.^29^

### Participation of racial and ethnic minorities in sentinel PARP inhibitor trials

Sentinel clinical trials for PARPi across ovarian, breast, prostate, and pancreatic cancers demonstrate persistent demographic and geographic disparities within the current landscape of precision oncology clinical trials. The evidence base supporting these trials is predominantly derived from non-Hispanic White populations, concentrating clinical data within a single demographic group (Table 1).

**Table 1.** Summary of population demographics from sentinel monotherapy PARPi trials across disease sites.

Disease site	Drug	Study	White (%)	Asian (%)	Black (%)	American Indian or Alaska Native (%)	Native Hawaiian or Other Pacific Islander (%)	Hispanic (%)	More than 1 race (%)	Other (%)	Missing (%)	Sample size
**Ovarian**	Olaparib	SOLO1^6^^,^^41^	81.8	15.1	1	0.3	1	NR	0.3	NR	1	*n* = 391
		SOLO2^30^^,^^42^	89.5	9.8	0.3	0	0	3.7	NR	0.3	NR	*n* = 295
	Rucaparib	ATHENA-MONO^8^	77.1	17.8	NR	NR	NR	NR	NR	3.2	1.9	*n* = 538
		ARIEL3^32^^,^^43^	77.3	3.7	1.4	0.7	0	NR	3.5	NR	13.3	*n* = 564
	Niraparib	NOVA^31^^,^^44^	86.8	3.4	1.3	0.2	0	NR	NR	NR	8.3	*n* = 553
		PRIMA^7^^,^^45^	89.4	3.4	1.6	0.1	0.1	NR	NR	NR	5.3	*n* = 733
**Breast**	Olaparib	OlympiAD^34^^,^^46^	65.2	31.1	1.7	1.3	0	NR	0	NR	0.7	*n* = 302
		OlympiA^33^^,^^47^	66.7	28.9	2.6	0.2	0.05	3.2	NR	0.5	1	*n* = 1836
	Talazoparib	EMBRACA^35^^,^^36^	69.1	10.9	2.8	0	0	10.7	0	17.2	NR	*n* = 431
**Prostate**	Olaparib	PROfound^37^^,^^48^	64.1	27.1	2.1	NR	NR	NR	NR	0.8	5.9	*n* = 387
	Rucaparib	TRITON2^38^^,^^49^	71.5	1.8	5.8	0	0.4	NR	NR	0.4	20.6	*n* = 277
		TRITON3^39^^,^^50^	74.6	1.2	3.5	0.2	0	1.7	0.7	NR	19.8	*n* = 405
**Pancreatic**	Olaparib	POLO 52^40^^,^^54^	91.6	3.9	3.2	0.6	0	3.9	0	NR	0.6	*n* = 154

Abbreviation: NR = not reported.

#### Ovarian cancer

SOLO1 was a landmark trial which established the efficacy of maintenance olaparib in high-grade serous or endometrioid ovarian, fallopian tube, or primary peritoneal cancer in patients with either a germline or somatic BRCA 1 or 2 pathogenic variant after a complete or partial response to first line platinum based chemotherapy, and led to the approval of olaparib monotherapy.^5^^,^^8^ SOLO2/ENGOT ov21 evaluated olaparib maintenance in recurrent platinum-sensitive ovarian cancer with germline BRCA mutations and led to a significant improvement in median PFS in this population.^30^ Of all the PARPi studies, these 2 demonstrated the overall lowest representation of Black participants.

PRIMA assessed the effectiveness of niraparib following a response to platinum-based chemotherapy expanding eligibility to include all patients at high risk for recurrence.^5^^,^^7^ The trial highlighted the most significant benefits for PARPi within subgroups with homologous recombination deficiency (HRD). NOVA/ENGOT ov16 evaluated niraparib in a population of 553 patients with platinum-sensitive recurrent high-grade serous ovarian, fallopian tube, or primary peritoneal cancer. In contrast to the SOLO2 study, NOVA including both germline BRCA (gBRCA) cohorts and non-BRCA cohorts.^31^ For both niraparib studies, the populations were predominantly White, and Hispanic ethnicity was not reported.

ATHENA-MONO/GOG-3020/ENGOT-ov45 explored the effectiveness of rucaparib versus placebo for extending PFS, regardless of genetic mutation status.^8^ ARIEL3/ENGOT ov21 confirmed maintenance rucaparib significantly prolongs PFS across BRCA-mutated, HRD-positive and overall intention-to-treat population. Compared to other PARPi trials in ovarian cancer, those with rucaparib had more significant representation from Asian patients.^32^

#### Breast cancer

In early-stage disease, OlympiA established that adjuvant olaparib for high-risk HER2-negative germline BRCA-mutated breast cancer after completion of local treatment and neoadjuvant or adjuvant chemotherapy was associated with significantly longer survival, free of invasive or distant disease, than placebo.^33^ The OlympiAD study, evaluating olaparib in metastatic HER2-negative germline mutated BRCA breast cancer, showed improved PFS with olaparib compared to physician’s choice chemotherapy.^34^

The EMBRACA study comparing talazoparib monotherapy to standard chemotherapy in patients with HER-2 negative advanced or metastatic breast cancer with germline BRCA1/2 mutations.^35^ All 3 studies had adequate Asian representation in their cohorts, but limited participation from Black and Hispanic patients. Median PFS was 5.6 months with the standard chemotherapy arm and 8.6 months with the talazoparib arm. Of the 431 enrollees, non-Hispanic White comprised 69.1%, Asian 10.9%, Black 2.8%, Hispanic 10.7%, Other 17.2%, and Missing not reported.^36^

#### Prostate cancer

PROfound was the first randomized, open-label phase III trial to demonstrate the importance of tumor genomic testing in prostate cancer and show improved radiographic progression-free survival (rPFS) with olaparib in homologous recombination repair (HRR) gene-mutated castration-resistant prostate cancer (mCRPC) compared with physician’s choice of enzalutamide or abiraterone.^37^ The study included patients with multiple Fanconi Anemia pathway mutations (Cohort A: BRCA1, BRCA2, or ATM mutations; Cohort B: 12 other HRR mutations).

The TRITON studies evaluated rucaparib in this population. TRITON2 was a phase 2, single-arm, open-label trial that evaluated rucaparib in BRCA1/2 mutated mCRPC.^38^

The follow-up TRITON3 was a phase 3, randomized, open-label trial in patients with chemotherapy-naive BRCA-mutated mCRPC that showed significantly improved rPFS of rucaparib compared to physician’s choice of therapy of docetaxel or second-generation androgen receptor pathway inhibitor.^39^ In both studies, the populations were predominantly White.

#### Pancreatic cancer

POLO was a phase 3, randomized, double-blind, placebo-controlled trial demonstrating that maintenance olaparib significantly improved progression-free survival in patients with germline BRCA1/2-mutated metastatic pancreatic cancer whose disease had not progressed after at least 16 weeks of first-line platinum-based chemotherapy but also in a predominantly White population.^40^^,^^51^

## Discussion

The lack of diversity in PARPi trials limits applicability of results across differing populations, and is driven by multiple factors, from study design to trial conduct. Variations in trial reporting, geographic sites of trial availability, restrictive eligibility, and access issues that ultimately impact drug dissemination all contribute to the current landscape.

### Systematic barriers to reporting

True trial diversity may be challenging to achieve due to logistical barriers that often oblige data homogenization, masking subgroup differences. General categories such as “Asian” or “Black” obscure immense ancestral and geographic diversity. For example, China leads PARPi research in the broader category of Asia, but these studies may overrepresent Chinese-descent enrollment as “Asian” when compared with studies run in other Asian countries.^52^ Collapsing broad categories without disaggregation, which has been shown to have prognostic value relative to cancer incidence and survival,^10^^,^^53^ contributes to limited diversity and representation. In addition, reporting of racial and ethnic categories such as Hispanic, American Indian, Alaska Native, and Pacific Islander are frequently omitted or relegated into a generic “Other” category. In fact, Taparra et al. noted in a systematic review that only 14% of Phase 2 or 3 clinical trial manuscripts in high-impact journals reported American Indian or Alaska Native race.^54^

As clinical oncology trials expand internationally, demographic reporting fragments further due to disparate legal and cultural principles governing racial identification.^55^ For instance, while global enrollment has modestly boosted Asian and Hispanic representation in oncologic clinical trials, Black participant enrollment remains persistently low. Despite oncology trials specifically including recruitment in Africa, enrollment remains depressed when compared to trials not specifically including Africa (3.2% including Africa vs 4.1% not including Africa),^55^ suggesting greater complexity to this observed underrepresentation.

In the PARPi trials discussed here, there was wide variation in reporting, with missing data common. Around 20% of the patients in TRITON 2 and TRITON3 had missing race/ethnicity data. Hispanic participant data were inconsistently reported, with the highest proportions in EMBRACA (10.7%, breast), TRITON3 (1.7%, prostate), and OlympiA (3.2%, breast). Nine of the 14 studies excluded Hispanic ethnicity from demographic reporting completely. Attention to comprehensive demographic data capture in future PARPi trials will be crucial to ensure thorough interpretation of study findings.

### PARPi trial geography

Current research on PARPis remains disproportionately concentrated in the Global North and high-income countries (HICs). Of the trials reported in Table 1, none had representation from the African continent, the Middle East (except for Israel), or the Indian subcontinent. While there were sites in South America, these were limited: PROfound had a site in Chile; SOLO1 had a site in Brazil; and OlympiAD had a site in Peru.^6^^,^^34^^,^^37^

Phase III randomized clinical trials (RCTs) are conducted predominantly in HICs (92%) and are more likely to be industry-funded (73%).^56^ A 2025 bibliometric analysis of 1434 publications on PARPis in ovarian cancer revealed numerous disparities of publication contribution.^52^^,^^57^ Top contributors to PARPi publications included the United States (440) and China (352). Other HIC’s included Italy (197), England (169), Spain (105), Japan (98), Germany (86), Canada (81), South Korea (78), Australia (65), and Denmark (50). All remaining countries/regions contributed fewer than 50 publications each. This geographic concentration represents resource disparities relative to need. Between 2016 and 2020, investment in LMICs accounted for only 0.5% of total global cancer research funding, despite roughly 80% of the world’s population residing in these regions.^58^^,^^59^ Sub-Saharan Africa bears a substantial share of the global cancer burden, yet only approximately 2% of all randomized clinical trials are conducted there. This results in a clinical research agenda that prioritizes cancers prevalent in wealthy nations, rather than matching the global burden of disease.^56^

In response to these geographic disparities, efforts to decentralize oncology clinical trials are expanding through dedicated regional organizations (Table 2).^9^ A recently opened trial (NCT06412120) evaluating the safety, tolerability, and efficacy of niraparib in patients of African ancestry with high-grade serous or high-grade endometrioid carcinoma of the ovary, fallopian tube, or peritoneum has established sites in Nigeria, the Bahamas, and the US to enhance ancestral and cultural diversity, and generate results with global applicability.^60^ Given the importance of characterizing safety in this population, the primary endpoint of the study is the proportion of women with and grade 3 or higher adverse event (AE), with an emphasis on the 11 most common AEs previously reported in the PRIMA study, especially thrombocytopenia and hypertension. Secondary endpoints include recurrence free survival, patient reported health-related quality of life, and population pharmacokinetics. Further exploratory analyses will seek to measure the prevalence of somatic homologous recombination deficiency in Black women; identify any single nucleotide polymorphisms predictive of differential drug metabolic activity; and characterize trends in circulating tumor DNA over the course of treatment. Studies inclusive of transcontinental populations such as this are important to ensure appropriate drug dosing and provide more personalized counseling about toxicity and management of side effects. Furthermore, demonstrating efficacy in previously unstudied populations allows increased advocacy for drug expansion in countries where PARPi may have limited availability and provides concrete evidence for the feasibility of conducting clinical trials in regions considered high risk.

**Table 2.** Regional and global organizations supporting the decentralization of oncology clinical trials.^9^

Region	Organizations
**Africa**	African Consortium for Cancer Clinical Trials (AC^3^T) Alliance for Accelerating Science in Africa (AESA) African Organization for Research and Training in Cancer (AORTIC) Human Heredity and Health in Africa (H3Africa)
**Latin America**	Latin American Cooperative Oncology Group (LACOG) Latin American Consortium for the Investigation of Lung Cancer (CLICaP) Grupo Argentino de Investigación Clínica en Oncología (GAICO) Grupo de Estudios Clínicos Oncológicos del Peruano (GECOPERU) Grupo Oncológico Cooperativo Chileno de Investigación (GOCCHI) The South West Oncology Group (SWOG) Latin America Initiative
**Asia**	Asian Clinical Trials Network for Cancers (ATLAS)
**Europe**	Immune Oncology Research Institute (IMMONC)
**Global policy and standards**	International Agency for Research on Cancer (IARC) National Institutes of Health (NIH) Global Alliance for Genomics and Health (GA4GH) International Council for Harmonization of Technical Requirements for Pharmaceuticals for Human Use (ICH)

### Eligibility criteria

A number of systemic issues in protocol design and conduct inadvertently exclude minority participation in trials. Standard exclusion criteria select against specific populations, especially when considering medical comorbidities and geographic disease distribution. Calculation of renal function and characterization of normal range; infectious status, such as HIV; and hereditary hematologic conditions, including sickle cell anemia and thalassemia, may exclude patients who are not otherwise high risk.^61^ These ailments are overrepresented in LMICs, the global South, and patients of African ancestry. For example, according to the World Bank, the prevalence of HIV in sub-Saharan Africa is 3%, the highest in the world.^62^ The highest rates of thalassemia are in Southeast Asia.^63^

A unique example of trial exclusion is benign congenital neutropenia, which disproportionately affects patients who are not of European ancestry. The Duffy null phenotype is protective against *Plasmodium vivax* and is found in 80%-100% of individuals from West Africa and 66.7% of those identifying as Black in the United States.^64^ The Duffy null-associated neutrophil count (DANC) reduces baseline absolute neutrophil count (ANC) by approximately 40% when compared to Duffy non-null individuals, without elevating infection risk.^64^^,^^65^ DANC contributes to the exclusion of individuals with African or Middle Eastern ancestry.^66^ Hibbs et al. reviewed 289 phase III trials and 71 curative-intent anticancer regimens for the 5 most prevalent cancers in the United States and United Kingdom. Of the trials reviewed, Hibbs et al. found 77% of the trials and 54% of the regimens excluded or modified doses for patients with neutrophil counts within the DANC range.^64^ In the PRIMA trial, neutropenia within the past year was an exclusion criteria, potentially limiting participation due to a low neutrophil count documented even before the start of cancer treatment.^7^

The American Society of Hematology advocates for the development of specific ANC reference ranges for individuals with the Duffy null phenotype to address systemic inequality in trial design and reduce structural barriers to clinical trial inclusion.^67^ Duffy null imposes well-documented structural barriers in the ANC criteria in clinical oncology trials, that disproportionately affect African/Middle Eastern ancestries, mirroring broader inequities.

### Global access

Equitable access to precision medicine reduces health disparities and enables comprehensive cancer care for all, but requires participation from global partners to ensure trial participants are reflective of their populations.^15^ The process from identification of patient eligibility to trial activation to ultimate drug approval by governing bodies, however, is lengthy and complicated. This can be dissuading to resource-limited regions, like the Global South, thus preventing such regions from being considered as potential sites.

International guidelines (NCCN/ESMO) endorse genetic and genomic testing for targeted treatments such as PARPi.^68^^,^^69^ In fact, this testing is now required for participation in trials of PARPi, and is crucial to data interpretation and risk stratification. Technical, infrastructure, and human capital constraints create a significant bottleneck in the implementation of clinical trials in LMICs as the logistics of genetic testing and result interpretation are burdensome. In regions such as Africa and South America, shortages of certified geneticists and counselors prevent timely results delivery and patient counseling.^70^ In addition, many LMICs lack in-country sequencing facilities and must rely on expensive international “send-out” testing, which inherently delays treatment.^71^

The regulatory burden is also significant, resulting in a decline in randomized clinical trials in LMICs, and thus decreased access.^72^ Clinical trials are de-prioritized in the setting of small healthcare budgets where capacity is strained and emphasis placed on different health issues (eg, water sanitation, infectious disease).^73^^,^^74^ Trial activation and oversight are limited by government policies intended to avoid exploitation, but become prohibitive.^75^ Complex oversight, layered approvals, and import restrictions may further delay trial activation and limits participation.^9^^,^^76^ Individuals in LMICs often lose access to the drug once a study concludes, raising additional ethical concerns about trial conduct in those locations.^77–79^ Even if these populations are included and efficacy is demonstrated, the time burden for new drug adoption in non-Western regions lags significantly behind HICs. For example, the time to regulatory approval for new drugs varies from 730 days post-FDA approval in Brazil to 1184 days in Colombia.^15^ Even following approval, these medicines face additional delays before becoming available in public healthcare systems, ranging from 156 days in Argentina to 1215 days in Mexico.^15^

The downstream financial toxicity is also significant. In HICs, the average out-of-pocket cost of PARPi averages $305/month, which is approximately 45% of total monthly health spend in an uninsured patient (Table 3).^80^ Conversely, in LMIC’s such as those in Latin America, monthly spending for PARPi treatments exceeds 15 days’ minimum wage.^81^ In addition, cancer drugs often carry higher prices in LMICs relative to gross domestic product or purchasing power.^15^ Pricing reductions alone are insufficient to ensure medication access without investment in diagnostic infrastructure and reimbursement models.^16^^,^^82^^,^^83^

**Table 3.** Diagnostic expenses and PARPi drug cost relative to annual income across geographic regions.^84–107^

Geographical region	Median annual household income (USD)	Estimated BRCA 1/2 cost (USD)^a^	Estimated PARPi cost per month (USD)
**North America (United States)**	53 400-84 000	0-400 (insured) 250-3000 (retail)	0-1200 (insured out of pocket cost) 1200-14 000 (median total cost)
**United Kingdom**	335 000	0-360	6000-9000 (list price for olaparib and niraparib)
**Germany**	55 000	0-250	6600 (pharmacy sales price)
**Australia**	62 600	0-1300	880-4600 (pharmacy sales price)
**Sub-Saharan Africa**	260-6100	100-800	2300 (estimated monthly single exit price) 4700 (pharmacy sales price)
**East Asia (India)**	2650	170-1200	70-6600 (pharmacy sales price)
**Asia-Pacific (China)**	13 660	110-650	1900-2100
**Latin America (Mexico, Brazil)**	9900-12 850	300-1600	3600-5000 (pharmacy sales price)

a Does not include the clinical consultation cost for interpretation, taxes, or send out costs.

## Conclusion

Sentinel PARPi trials across ovarian, breast, prostate, and pancreatic cancer demonstrate nearly universal inclusion of primarily non-Hispanic White populations. Black, Asian, Hispanic, and other groups are significantly underrepresented relative to known distribution of global cancer burden. This creates significant limitations in data interpretation and applicability of results across diverse populations relative to impacts on survival, toxicities, and quality of life, and is especially relevant as work is conducted to enhance access to global populations who will benefit from these drugs. Homogenous study populations, however, are representative of larger systemic issues in the conduct of clinical trials, including accurate reporting of participants, diverse selection of sites across geographies, and more inclusive protocol eligibility. Continuing to objectively observe and address the multifaceted barriers to trial enrollment and reporting, as well as creating more robust global infrastructure for research and regulatory approvals, will be crucial as the oncology community seeks to ensure widespread health equity in cancer care.
